# Dislocation of the Femur into the Obturator,—Reduction by a Block between the Thighs, after the Pulleys and Jarvis’ Adjuster Had Failed

**Published:** 1852-03

**Authors:** Daniel Brainard

**Affiliations:** Prof. of Surgery in Rush Medical College, etc.; Chicago


### ARTICLE X.

*Dislocation of the Femur into the Obturator Faramen. Reduction by a block between the Thighs after the Pulleys and Jarvis' Adjuster had failed.* By DANIEL BRAINARD, M. D., Prof. of Surgery in Rush Medical College, etc.

Sunday, Feb. 23, 1852, I was requested to visit Michæl Sweeney near Michigan City, in Indiana, on account of a dislocation of the femur, which had occurred the Friday previous.

Feb. 20. I arrived there on the evening of Monday, 23d ; I found a dislocation of the right femur into the obturator foramen with all the signs of that accident extremely well marked. Efforts at reduction had at first been made by the attending Physician without success. Subsequently the compound pulleys had been resorted to and this not being effectual, "Jarvis' Adjustor" was employed without any better result. As these means had been used by Prof. Daniel Meeker, late of the Indiana Medical College, to whose politeness I was indebted for the call to visit the case, I presumed that more efforts by such means were not advisable and accordingly sought for others.

I had for some time been of the opinion that a wedge between the thighs kept close to the perinæum and sufficiently large to fill up the space, might be made use of as a fulcrum and the members themselves employed as levers by which that accident could be remedied.

At first the right femur was directed so much outward as to prevent this from being employed to advantage,and the pulleys were applied at the ankle to make extension nearly in the line of the axis of the body. Having continued this moderately about 20 minutes, until the members were more nearly in a parallel position, I placed a stick of wood about 4 inches in diameter wrapped about with several layers of a wadded quilt, between the thighs, and relaxing the pulleys, siezed one of the legs, while Dr. Everett, who rendered efficient assistance, held the other, and by simply pressing them together, taking care to keep the knees straight, the bone went into its socket with a loud snap.

This form of dislocation is said to be the least difficult of reduction of all those of the hip, but it is probable that there are exceptional cases where a variety of means might be desired. In such the method used in this would be applicable, and it is not improbable that it might be as well to resort to it in the first instance.

*Chicago, Feb.* 26, 1852.

